# Six Glycolysis-Related Genes as Prognostic Risk Markers Can Predict the Prognosis of Patients with Head and Neck Squamous Cell Carcinoma

**DOI:** 10.1155/2021/8824195

**Published:** 2021-02-10

**Authors:** LangXiong Chen, XiaoSong He, ShiJiang Yi, GuanCheng Liu, Yi Liu, YueFu Ling

**Affiliations:** ^1^Otolaryngology & Head and Neck Surgery, The Affiliated Hospital of Guilin Medical University, Guilin, Guangxi Zhuang Autonomous Region, China; ^2^The Guilin Medical University, Guilin, Guangxi Zhuang Autonomous Region, China

## Abstract

**Objective:**

Head and neck squamous cell carcinoma (HNSCC) is one of the worst-prognosis malignant tumors. This study used bioinformatic analysis of the transcriptome sequencing data of HNSCC and the patients' survival and clinical data to construct a prediction signature of glycolysis-related genes as the prognostic risk markers.

**Methods:**

Gene expression profile data about HNSCC tissues (*n* = 498) and normal tissues in the head and neck (*n* = 44) were got from The Cancer Genome Atlas (TCGA), as well as patients' survival and clinical data. Then, we obtained core genes; their expression in head and neck squamous cell carcinoma tissues is significantly different from that in normal head and neck tissues. The predicted glycolysis-related genes are screened through univariate Cox regression analysis, and then, the prognostic risk markers were constructed through further correction of multivariate Cox regression analysis. The Kaplan-Meier curve and receiver operating characteristic curve are used to analyze the potential value of these risk markers in diagnosis and prognosis. We also evaluated that the glycolysis-related prognostic risk markers composed of 6 oncogenes are correlated with clinical features, such as age, gender, grade, and clinical stage of the tumor, by univariate and multivariate Cox regression analyses.

**Results:**

Differentially expressed glycolytic genes in HNSCC tissues and normal head and neck tissues were screened from TCGA databases using the bioinformatic method. We confirmed a set of six glycolytic genes that were significantly associated with OS in the test series. According to our analysis, the prognostic risk markers composed of HPRT1, STC2, PLCB3, GPR87, PYGL, and SLC5A12 may be an independent risk factor for the prognosis of HNSCC.

**Conclusions:**

Through this analysis, we constructed new prognostic risk markers related to glycolysis as a prognostic risk marker for patients with HNSCC and provided new ideas and molecular targets for the research and individualized treatment of HNSCC.

## 1. Introduction

Head and neck cancer is one of the main causes of global morbidity and mortality, of which HNSCC accounts for 90% [1]. Current research indicated that HNSCC is closely related to numerous factors, including smoking, drinking, and human papilloma virus [2, 3]. In recent years, fiber rhinopharyngoscope and other related examinations have been widely applied to detect early lesions of HNSCC; hence, HNSCC patients can be diagnosed and treated early [4]. In order to study the mechanism of HNSCC, countries from all around the world have invested a lot in scientific research and made great progress in diagnosis and treatment of HNSCC [5]. However, the incidence and mortality of HNSCC remain high [6]. In addition, due to the heterogeneity of molecular mechanisms and tumor behaviors related to HNSCC, the widely recognized clinical factors including lymph node metastasis and histological grade are not sufficient to predict the prognosis of a patient. Therefore, identifying clinically relevant biomarkers for HNSCC can facilitate correct treatment decisions and improve prognosis [7]. However, studies have not identified an effective clinical biomarker for the prognosis of HNSCC so far.

Aerobic glycolysis is one of the most important features of tumor metabolism [8]. In recent years, research has found that this feature can be a new breakthrough for studying tumor occurrence and development and antitumor treatment. An increasing number of studies have found that glycolysis-related genes may make for prognostic risk markers [9], but most of the markers have not been clinically applied, and studies that select and analyze a specific characteristic or a single signal pathway are rare. This may be a new key breakthrough for our research on the occurrence and development of tumors. This study intends to set up a prediction signature of glycolysis-related genes as the prognostic risk markers by mining the transcriptome sequencing data of HNSCC.

## 2. Materials and Methods

### 2.1. Obtained HNSCC Gene Expression Profile and Clinical Related Data from TGCA

The transcriptome gene expression profile of HNSCC was obtained from TCGA (https://portal.gdc.cancer.gov/repository), and the corresponding clinical follow-up information was also obtained from TCGA. Then, we obtained a total of 498 cases of HNSCC patients and 44 cases of normal tissue gene expression profiles and corresponding clinical information.

### 2.2. Identified Prognostic Glycolysis-Related Genes and Constructed New Predicted Risk Markers

Gene Set Enrichment Analysis (GSEA) is used to detect 4 gene sets about glycolysis (GO_LACTATE_TRANSPORT, CHEN_LUNG_CANCER_SURVIVAL, HALLMARK_GLYCOLYSIS, and REACTOME_GLYCOLYSIS) from GSEA (https://www.gsea-msigdb.org/gsea/login.jsp); the gene files of gmp format were got from the Molecular Signatures Database (https://www.gsea-msigdb.org/gsea/msigdb/index.jsp). Then, we analyzed the expression differences of the above-mentioned glycolysis-related gene set in HNSCC tissues and normal tissues in the head and neck. The expression differences of glycolysis-related genes were characterized by associated *p* values and logFC (fold change), and the core genes was constructed. The risk markers related to prognosis were screened by univariate and multivariate Cox regression analyses based on the core genes, and the prognostic risk markers were constructed.

### 2.3. Statistics and Survival Analysis

The screening of differential genes and the analysis of the Cox proportional hazards regression model in this research are mainly achieved by R language (R x64 3.6.3). We loaded the limma (Linear Models for Microarray Data) package to analyze the differential expression of the preprocessed data.

Sorting out the gene expression information through linear models, a comparison model was constructed to compare the gene expression data of patients in the high-risk and low-risk groups. These patients' unique risk score was obtained based on the prognostic risk score formula and the expression level. Risk score = expression of gene 1 × *β*1 + expression of gene 2 × *β*2 + ⋯+expression of gene *n* × *βn*. A unique risk score for each patient with HNSCC was divided into the high- and low-risk groups by the median of risk score values.

A Kaplan-Meier curve is used to analyze the constructed prognostic risk markers to predict the patients' OS with HNSCC. When using prognostic risk markers to predict a patient's OS, their diagnostic efficacy is further verified through receiver operating characteristic (ROC) curve analysis. The relation between the risk score and clinical characteristics was analyzed by univariate and multivariate Cox regression analyses, including age, lymph node metastasis, stage, gender, and classification.

*p* value < 0.05 was considered statistically significant.

## 3. Results

### 3.1. Glycolysis-Related Gene Sets

**Figure 1:**
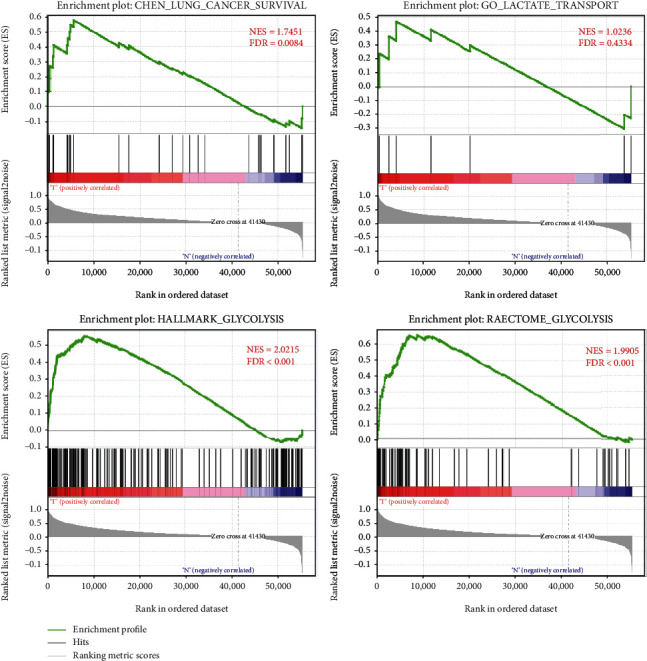
GSEA of glycolysis-related gene sets. Enrichment plots of four glycolysis-related gene sets between HNSCC and paired normal tissues identified by GSEA.

This study analyzed HNSCC such as oral squamous cell carcinoma and throat squamous cell carcinoma. The transcriptome expression profile of HNSCC can be obtained from TCGA, and the corresponding clinical follow-up data can also be obtained from TCGA. We observed and screened the glycolysis-related gene sets on the GSEA website. We, respectively, explored 4 different gene sets (CHEN_LUNG_CANCER_SURVIVAL, GO_LACTATE_TRANSPORT, HALLMARK_GLYCOLYSIS, and REACTOME_GLYCOLYSIS) as to whether the expression of tumor samples in these 4 glycolysis-related gene sets is different from that of normal head and neck samples. We found that the CHEN_LUNG_CANCER_SURVIVAL, REACTOME_GLYCOLYSIS, and HALLMARK_GLYCOLYSI gene sets in HNSCC are significantly different between the normal head and neck samples and the tumor samples (FDR are 0.008, <0.001, and <0.001, respectively). For HNSCC, the GO_LACTATE_TRANSPORT gene set is not significantly different between the tumor sample and the normal head and neck sample (FDR = 0.433) (Figure 1). Next, the core genes were screened from 3 glycolysis-related gene sets (CHEN_LUNG_CANCER_SURVIVAL, HALLMARK_GLYCOLYSI, and REACTOME_GLYCOLYSIS), which are the differentially expressed genes about glycolysis in the tumor tissue and normal tissue, as shown in Table 1.

**Figure 2:**
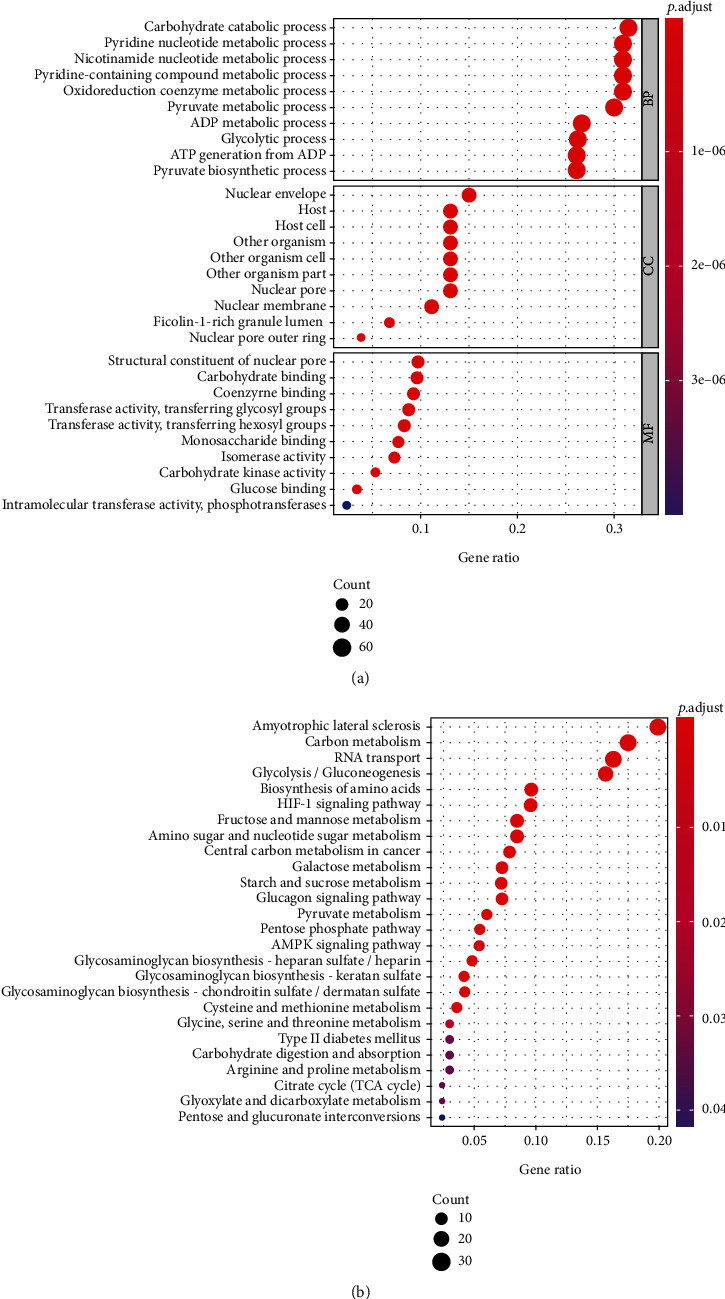
The functional enrichment analysis of the core genes. Gene Ontology (GO) terms (a) and Kyoto Encyclopedia of Genes and Genomes (KEGG) pathways (b) were significantly enriched by the core genes.

We further verified the correlation between core genes and glycolysis; GO analysis is used to evaluate their biological processes (BP), molecular function (MF), and cellular component (CC). Next, KEGG pathway enrichment analysis is used to verify their biological pathways. The results show that the biological processes (BP) of these core genes have the highest enrichment among several metabolic processes, molecular function (MF) is related to the enzyme activity and glucose binding of multiple metabolic pathways, and the KEGG pathway enrichment analysis involves carbon metabolism and glycolysis/gluconeogenesis. It was further verified that the core genes screened out were related to glycolysis (Figure 2).

### 3.2. Construction of Prognostic Risk Markers Based on Core Genes

The overall survival data of patients with HNSCC were sorted out, and these core genes were further analyzed. The glycolysis-related genes related to the patient's OS were screened through univariate Cox regression analysis. The correlation of glycolytic gene expression profile and patient's OS was further verified though multivariate Cox regression analysis. After analysis, the screened result is the prognostic risk markers composed of OS-related glycolysis genes, that is, HPRT1, STC2, PLCB3, GPR87, PYGL, and SLC5A12. The prognostic risk markers based on glycolysis-related oncogenes are constructed (Table 2).

**Figure 3:**
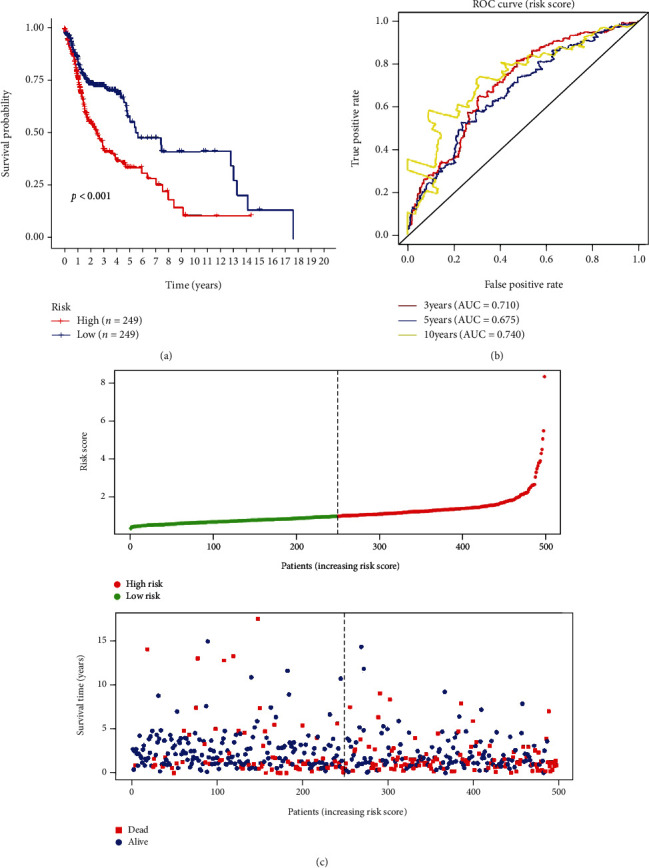
Risk score based on the 6 glycolysis-related gene prognostic risk markers in patients with HNSCC. (a) Kaplan-Meier curve of OS in the high- and low-risk groups. (b) Time-dependent ROC curves of the 6 glycolysis-related gene signatures for prediction of 3-, 5-, and 10-year OS. (c) The distribution of the 6 glycolysis-related gene risk scores and survival status for each patient.

The regression coefficient (*β*) of each prognostic risk marker was calculated through multivariate Cox analysis. These patients' unique risk score was obtained based on the regression coefficient and the expression level. Risk score = expression of HPRT1 × 0.019464 + expression of STC2 × 0.0261 + expression of PLCB3 × 0.012804 + expression of GPR87 × 0.005647 + expression of PYGL × 0.005313 + expression of SLC5A12 × 0.384181. A unique risk score for each patient with HNSCC was divided into the high- and low-risk groups by the median of risk score values and then the high-risk (*n* = 249) and low-risk (*n* = 249) groups, respectively. The Kaplan-Meier (KM) survival curve was used to evaluate the prognosis difference between both groups. Results show that the survival rate of the high-risk group is significantly lower than the low-risk group (*p* value < 0.001) (Figure 3(a)). In order to test the impact of glycolysis-related prognostic risk markers composed of six oncogenes in predicting the 3-, 5-, and 10-year OS rate of HNSCC patients, their diagnostic efficacy was further verified by ROC curve analysis. The results found that the area under the curve (AUC) is 0.710, 0.675, and 0.740, respectively (Figure 3(b)), indicating that the prognostic risk markers have a good performance in predicting the OS rate of HNSCC patients. Based on the risk scores, the risk curve was constructed (Figure 3(c)); we further found that the higher the risk score, the less the patients' survival time.

### 3.3. The Prognostic Risk Markers and Clinical Characteristics

**Figure 4:**
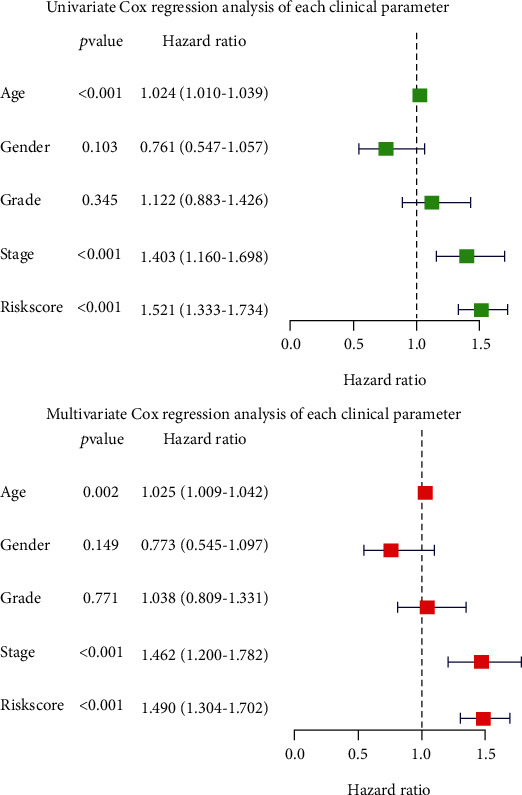
Univariable and multivariable analyses for the risk score and each clinical feature.

In order to assess whether the glycolysis-related prognostic risk markers composed of 6 oncogenes are correlated with clinical features such as age, gender, grade, and clinical stage of the tumor, we used clinical parameters as covariates in the entire data set with univariate and multivariate Cox regression analyses. The result data shows that in the univariate Cox regression analysis, age, tumor clinical stage, and risk score are both significantly correlated with overall survival time, while gender and tumor grade are not significantly correlated. After multivariate adjustment, the risk score is still significantly correlated with the overall survival time (*p* < 0.001, 95% CI 1.304-1.702, HR = 1.884). The result of univariate and multivariate Cox regression analyses and stratified analysis shows that the glycolysis-related prognostic risk markers composed of the six screened oncogenes predict patient prognosis and can be independent of clinical characteristics. In a word, the prognostic risk markers could be applied to predict OS in patients with HNSCC (Figure 4).

**Figure 5:**
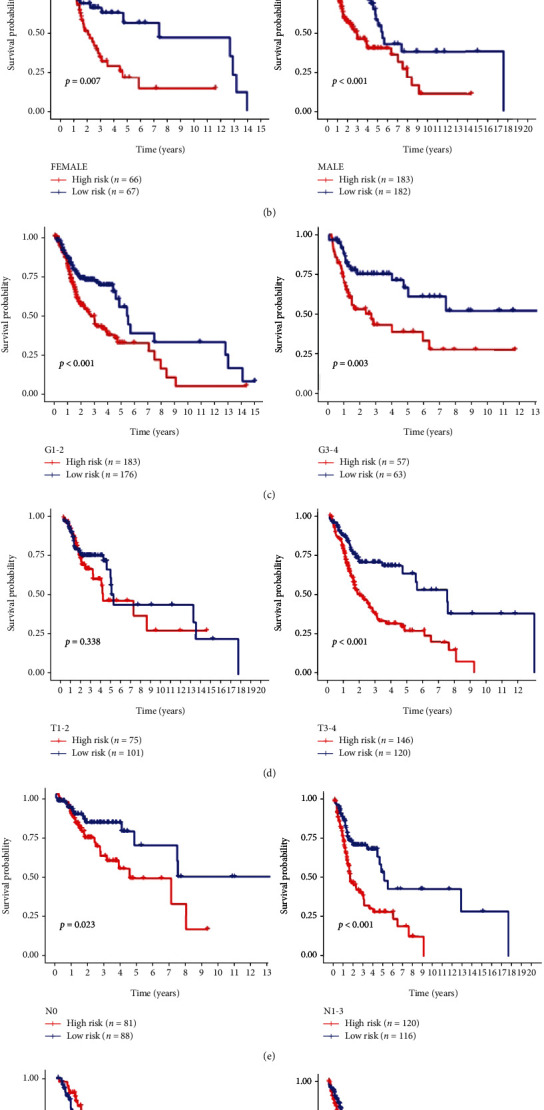
Stratification analysis of various clinicopathological factors by Kaplan-Meier curves for the patients with HNSCC in the TCGA dataset. Kaplan-Meier curves of OS in different subgroups stratified by (a) age, (b) gender, (c) grade, (d) T stage, (e) N stage, and (f) AJCC stage.

We further evaluate the risk score correlation with clinical characteristics, excluding patients with unknown clinical data (including age, gender, grade, T, N, and tumor clinical stage). We performed a stratified analysis of each clinical characteristic, respectively. The prognostic risk markers stratified patients based on age; one is the ≤65-year-old group with high risk (*n* = 159) and low risk (*n* = 165), and the other is the >65-year-old group with high risk (*n* = 90) and low risk (*n* = 84). It is concluded that the overall survival rates between the two subgroups in both groups have significant differences (*p* < 0.001 and 0.003, respectively). The patients were stratified by gender and are divided into males and females. Among them, women have 66 high-risk cases and 67 low-risk cases; men have 183 high-risk cases and 182 low-risk cases. The analysis shows that the overall survival rates between two subgroups in both groups are significantly different (*p* = 0.007 and <0.001, respectively). Then, all patients were stratified based on the tumor grade, divided into two subgroups, named grades I and II (*n* = 359) and grades III and IV (*n* = 120), respectively. Based on the rick score, grades I and II have 183 high-risk cases and 176 low-risk cases. We found that overall survival of the two subgroups is significantly different (*p* < 0.001). The same results are also observed in two subgroups of grade III and IV patients (*p* = 0.003). Based on the T stage of tumors, patients were divided into the T1 and T2 groups (*n* = 176) and the T3 and T4 groups (*n* = 266). Among them, the survival rate of high-risk patients in the T1 and T2 groups is not significantly different from that of the low-risk subgroup (*p* = 0.338), while the survival rate of high-risk patients in the T3 and T4 groups is significantly different from the low-risk subgroup (*p* < 0.001). According to the presence or absence of lymph node metastasis, these patients were divided into two groups. We found that survival rates between high-risk and low-risk patients in both groups are significantly different (*p* = 0.023; *p* < 0.001). Lastly, we divided patients based on the clinical stage of the tumor: one is the stage I and II group (*n* = 94) with high risk (*n* = 45) and low risk (*n* = 49) and the other is the stage III and IV group (*n* = 336) with high risk (*n* = 174) and low risk (*n* = 162). In the stage III and IV group, the survival rate of high-risk patients is significantly different from low-risk patients (*p* < 0.001). In another group, *p* = 0.700 indicates that there is no significant difference in the survival rate of patients in the two subgroups (Figures 5(a)–5(f)).

### 3.4. Expression of Six Glycolysis-Related Genes in HNSCC

**Figure 6:**
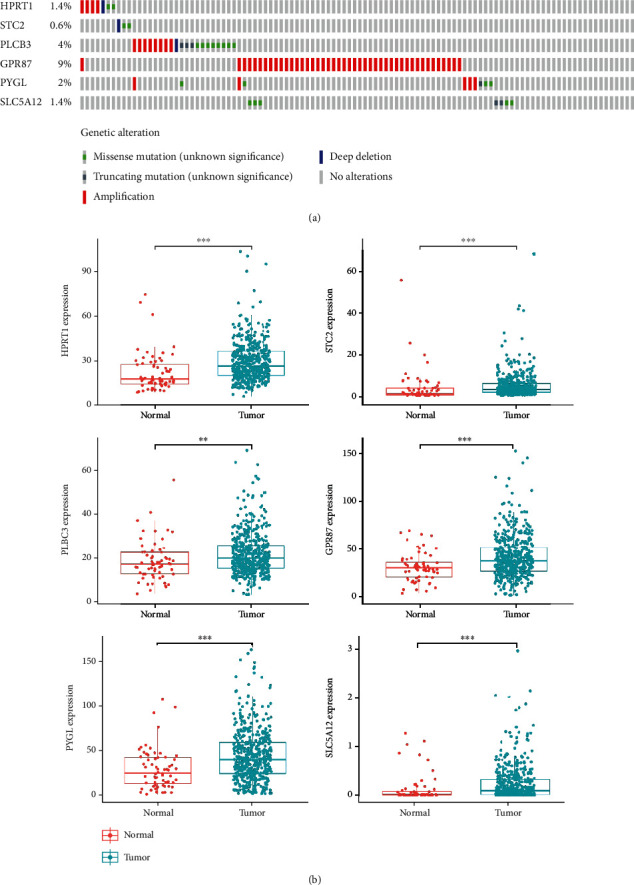
Analysis of the expression of 6 genes in the sample (a) to screen the mutations of genes in sample of *M* cancer patients (b); the differential expression analysis of the 6 screened genes (∗means < 0.05, ∗∗means < 0.01, and ∗∗∗means < 0.001).

In the cBioPortal online database, we analyzed the mutations of 6 glycolysis-related genes in HNSCC through clinical samples. The result shows that in overall 498 cases, 93 patients (18.6%) had gene mutations. Among them, HPRT1 gene mutations include 4 cases of amplification, 2 cases of missense mutations, and 1 case of deep deletion; STC2 gene mutations account for 0.6%, PLCB3 gene 4%, GPR87 gene 9%, PYGL gene 2%, and SLC5A12 gene 1.4% (Figure 6(a)).

**Figure 7:**
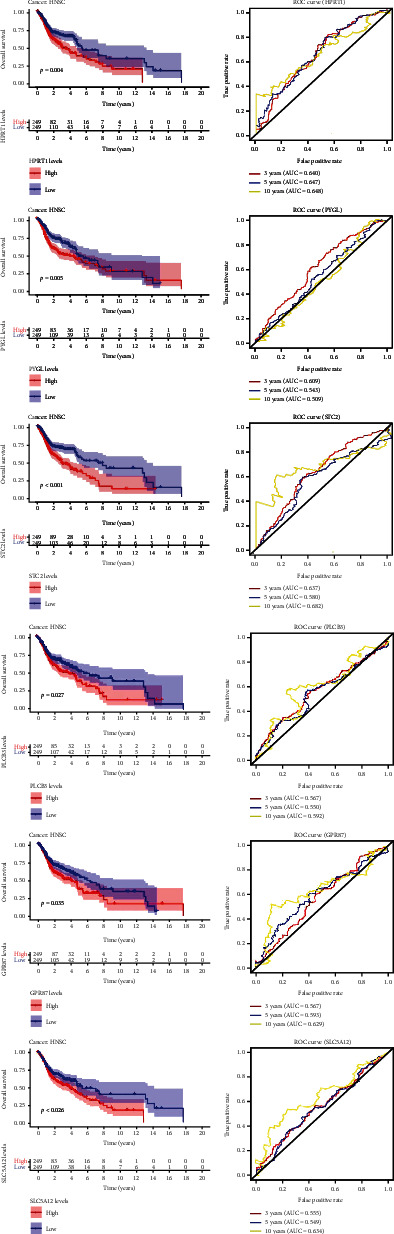
The expression based on each glycolysis-related gene predicts OS in patients with HNSCC and Kaplan-Meier curve of OS in the high- and low-expression groups. (b) Time-dependent ROC curves of each glycolysis-related gene for prediction of 3-, 5-, and 10-year OS.

We further analyzed the differential expression of HPRT1, STC2, PLCB3, GPR87, PYGL, and SLC5A12 in HNSCC and normal head and neck tissue and found that the expression of 6 glycolysis-related genes in HNSCC tissues is significantly upregulated compared with normal tissues (among which *p* < 0.001 for HPRT1, STC2, PLCB3, GPR87, and SLC5A12; *p* < 0.01 for PYGL) (Figure 6(b)). We sorted the expressions of each gene in the tumor samples from HNSCC patients, and then, these patients were split into two subgroups based on the median expression value, high- and low-expression groups, respectively. The Kaplan-Meier curve analyzes whether the high or low expression of each gene is correlated with the OS of HNSCC patients. It is found that HPRT1, STC2, PLCB3, GPR87, PYGL, and SLC5A12 may be correlated with the poor prognosis of HNSCC (*p* = 0.004, <0.001, 0.027, 0.035, 0.030, and 0.009, respectively) (Figure 7(a)). When the HPRT1, STC2, PLCB3, GPR87, PYGL, and SLC5A12 were used as independent biomarkers, their prognostic efficacy needs further verification. We performed ROC curve analysis further which verified their prognostic efficacy (Figure 7(b)), but the AUC values of the six glycolysis-related genes predicting OS of HNSCC patients were all less than 0.710, 0.675, and 0.740, respectively, and their predictive performance was all worse than prognostic risk markers.

## 4. Discussion

The active proliferation, differentiation, and other life activities of cancer cells are significantly not like the normal ones [10]. In order to provide the large quantity of protein, lipid, nucleic acid, and other molecular materials that tumor cells need, the proliferation and metastasis of malignant cells require metabolic reprogramming to accelerate anabolism to support cell growth [11]. In order to meet its biosynthetic needs, tumor cells increase glucose uptake through glycolysis and oxidative phosphorylation. A series of changes make tumor cells reregulate the production of ATP or increase glucose uptake to facilitate energy production. Studies have shown that with adequate oxygen, tumor cells can promote rapid energy supply of glycolysis due to the change of microenvironment, which is known as the “Warburg Effect” [12]. In the “Warburg Effect,” a tumor cell promotes glycolysis to produce sufficient ATP to maintain its proliferation and differentiation [13]. At the same time, lactic acid, the end product of glycolysis, is released into both internal and external of the cell, acidifying its microenvironment and facilitating the formation of microcapillaries. This process helps tumor cells to meet their nutritional requirements and strengthen their resistance to oxidative damage, which may also be an important immune escape mechanism in tumors [14].

According to reports, when clinicians predict the prognosis of cancer patients, traditional clinical parameters and pathological characteristics are not enough to accurately predict. The comprehensive genomics research based on high-throughput ribonucleic acid sequence and microarray map has made significant research progress, and some research results have been developed and applied to clinical practice [15]. Some biomarkers used to predict the prognosis of cancer patients have been confirmed, such as glycolysis-related genes. Although the specific mechanism between glycolysis and metastasis in the process of cancer progression is still unknown, more and more glycolysis-related genes were verified that are related to the development, differentiation, invasion, and metastasis of cancer cells in recent years. A variety of glycolysis-related genes are related to regulating cell metabolism to maintain tumor proliferation, metastasis, and invasion. Yin et al. [16] found that 4-mRNA signature related to glycolysis may be prognosis marks of patients with bladder cancer and may correlate with the occurrence and development of tumor cells. In addition, Chen et al. [17] found that glycolysis-based seven genes were identified, which can be used as a prognostic marker for patients with colon adenocarcinoma. Meanwhile, the progress achieved in tumor glycolysis and metabolism has promoted rapid development of personalized cancer treatment and management [18]. The identification of key biomarkers may help to formulate personalized treatment and prognosis of HNSCC. Although surgical resection is still an important treatment for HNSCC patients, increasing adjuvant treatments (such as chemotherapy, radiotherapy, and immunotherapy) have achieved rapid development in recent years, especially for patients with advanced HNSCC. Due to the heterogeneity of HNSCC and its resistance to chemotherapy, it is still a challenge for clinicians and pathologists to predict high-risk HNSCC and on how to perform precise treatment. Therefore, reliable prognostic markers are urgently needed to guide the treatment options for patients with HNSCC. In this study, by analysis of overall survival of patients with HNSCC by the Kaplan-Meier curve, we noticed that the glycolysis-related prognostic risk markers composed of HPRT1, STC2, PLCB3, GPR87, PYGL, and SLC5A12 can be applied as a predictive biomarker for the prognosis of HNSCC patients. Subsequent univariate and multivariate Cox proportional hazards regression analyses indicated that the risk score is significantly correlated with patients' OS, suggesting that the prognostic risk markers of glycolysis-related genes predict patient prognosis and can be independent of clinical characteristics. And then, we analyzed that HPRT1, STC2, PLCB3, GPR87, PYGL, and SLC5A12 are highly expressed in HNSCC tissues; besides, HPRT1, STC2, PLCB3, GPR87, PYGL, and SLC5A12 may be related to the poor prognosis of HNSCC. This study constructs prognostic risk markers related to glycolysis as a prognostic marker for patients with HNSCC, providing new ideas and molecular targets for the research and individualized treatment of HNSCC.

Our result indicates that the prediction signature composed of glycolysis-related genes has shown potential in predicting prognosis and personalizing treatment of patients with HNSCC.

## 5. Conclusion

In this study, there are certain limitations in our research. Due to the retrospective characteristics, this study may lead to selection bias. The sample size in the validation data set is insufficient. Therefore, this prediction model needs more prospective clinical trials for further verification. In addition, the related mechanisms by which glycolysis-related genes regulate HNSCC need further study. We first identified and verified the prognostic risk markers composed of six glycolysis-related genes, that is, HPRT1, STC2, PLCB3, GPR87, PYGL, and SLC5A12. These prognostic risk markers can predict the prognosis of patients with HNSCC, suggesting that it can be used as a prognostic risk marker for HNSCC. We provided new insights into the correlation between glycolysis and HNSCC. These risk markers may be a valuable prognostic indicator in clinical practice, helping to identify patients with HNSCC with poor prognosis. New ideas were provided by our results for studying the evolution mechanism of HNSCC and its individualized treatment.

## Figures and Tables

**Table 1:** The core gene were screened from 3 glycolysis-related gene sets.

The ID of core gene (*n* = 207)
DLD, NUP35, NUP188, HPRT1, IL13RA1, B4GALT7, PEBP1, NUP54, NUP205, GPI, STC2, NUP214, SL16A8, MDH2, TGFBI, NDC1, NUP58, CD44, PGP, PPIA, AGL, SERPINA1, TFF3, HSPA5, RBCK1, PPP2R5D, SLC16A3, B4GALT4, ARPP19, PKM, EXT1, B4GALT1, UGP2, XYLT2, KRT7, PFKFB1, AGRN, PGM2L1, NUP160, POLR3K, SLC16A7, SDC3, PFKP, STC1, ABCB6, NUP155, NDUFV3, B3GAT3, BPNT1, NUP88, P4HA1, TXN, CENPA, NASP, PMM2, BPGM, NUP37, PLCB3, KIF2A, NUP133, HMMR, ISG20, EGLN3, CXCR4, GYS2, GCKR, NUP107, CDK1, GOT1, HK3, SDC1, ATP5F1D, MED24, CASP6, PGK1, SLC16A1, CLN6, GCK, HS2ST1, PFKL, VCAN, HK1, GNPDA1, LDHA, ELF3, B4GALT2, MXI1, CACNA1H, EXT2, GMPPA, CLDN3, ANXA1, ARTN, SLC5A8, PAXIP1, CHPF, GPR87, NANP, PDK3, GAPDHS, NUP62, COG2, SOD1, SDC2, NT5E, PLOD2, NUP50, PFKFB4, PRPS1, TPST1, COPB2, GCLC, GLCE, SAP30, PC, SPAG4, GALE, NUP85, ECD, POM121, ANG, NUP98, IGFBP3, CHST4, CHST12, GNE, PYGL, PSMC4, P4HA2, SLC25A13, PFKFB3, ALDH7A1, PLOD1, GRK4, KIF20A, TPBG, LCT, HK2, MERTK, CTH, PPP2CB, NUP153, AKR1A1, EPHX1, AK3, PYGB, ACTB, DH1, G6PD, RAE1, ALG1, MIOX, GPC1, TPR, GSTP1, STMN1, ME1, CYB5A, FBP2, EGFR, NUP210, ENO1, ENO3, SLC5A12, KDELR3, GALK2, B3GALT6, COL5A1, FUT8, PGM2, AAAS, CAPN5, CHST2, PGAM1, ADPGK, LHPP, ADORA2B, FKBP4, VLDLR, RRAGD, GAPDH, SEH1L, NUP93, RPE, CHPF2, DEPDC1, DDIT4, ENO2, ME2, CITED2, VEGFA, TGFA, NUP43, AURKA, CHST1, ALDOC, LHX9, BIK, MET, GUSB, CALU, ANKZF1, ALDH9A1, POM121C, TPI1, GMPPB, MIF.

**Table 2:** The information of six glycolysis-related genes associated with overall survival in patients with HNSCC.

Gene	Cox (*β*)	HR
HPRT1	0.019464	1.019654
STC2	0.0261	1.026443
PLCB3	0.012804	1.012886
GPR87	0.005647	1.005663
PYGL	0.005313	1.005327
SLC5A12	0.384181	1.468411

## Data Availability

All the data and information used in the article are from public websites, including TCGA (https://portal.gdc.cancer.gov/repository), GSEA (https://www.gsea-msigdb.org/gsea/login.jsp), and the Molecular Signatures Database (https://www.gsea-msigdb.org/gsea/msigdb/index.jsp). Data sharing is not applicable to this article.
